# Enteric Fever in South Africa

**Published:** 1901-02-09

**Authors:** 


### The Hospital

A Journal of

The Medical Sciences and Hospital Administration.

Vol. XXIX.—No. 750.] Edited by Sir Henry Burdett, K.C.B., and Solomon C. Smith, M.D., M.R.C.P. [February 9, 1901.

### Enteric Fever in South Africa.

Now that the time is, to all appearance, approaching when Lord Kitchener will be sufficiently supplied with mobile troops to commence effective proceedings against the Boers who are maintaining in South Africa a condition of active war, although apparently devoid of any central or other government, the public are not unnaturally becoming anxious that every possible care should be taken of the lives of the men who will be engaged in a dangerous form of service. It is becoming evident that we are on the verge of momentous issues. The northern and the western portions of Cape Colony are occupied by a disaffected population, ready to rise against us on the slightest sign of success on the part of the enemy; and in the opinion of many who profess to know the drift of affairs in that part of the world, the issue which must be fought out and decided within the next few weeks is the most serious of any that we have had to face since the early days of the war, when, but for the stand made at Ladysmith, the whole of our colonies might have been crushed down on to their sea-ports. At the present moment, then, mobility is worth everything to us, and we cannot but see that nothing fetters the movements of an army more than the chain of sickness by which so far our armies have been tied fast to their bases. It is impossible, then, to avoid anxiety in regard to the recent increase in the prevalence of typhoid fever in South Africa, especially as it takes place at the recurrence of the season during which the prevalence of enteric fever last year was of such a kind as seriously to cripple the operations of the Commander-in-Chief, and to occasion the loss of a large number of valuable lives, that of the Queen's grandson among others. The average gentleman who stays at home at ease, and who from that safe retreat instructs the readers of the newspapers which he honours with his correspondence as to the methods by which perfect health might be secured amid the stress of field service, has not been wanting to his country in the emergency. He, like Voltaire's "Frère Lourdis"—

> "riant d'un sot rire,

> Ne comprend rien, et toute chose admire."

He is full of strange suggestions, but the point of real importance is to know whether the chief officers of the Medical Department have been called to council by the authorities of the War Office, and whether their recommendations, founded upon the actual experience of last year, have as far as possible been adopted.

It is well known that enteric fever, dysentery, and other maladies chiefly affecting the intestinal tract, have always been among the most fatal scourges of great armies; and nothing tells more strongly of the advances of sanitary science than the degree in which the losses from these scourges have been diminished. According to some figures recently published, the disease mortality among the troops in South Africa, if it had continued for a year at the same level which it attained at the end of May, would have amounted to an *annual* death rate of 94 per 1,000. In the Crimea, during the *one month* of January, 1855, 10 per cent. of the whole English invading force actually perished from disease. There can be no question that the health of the soldier has been infinitely better cared for during the present war than it was during the last great struggle in which we were engaged, and that the care has rewarded those by whom it has been taken. Nor, we think, can there be any question that still more might be accomplished in the same direction, but it is less certain by what means. We know that the microbe of enteric fever will grow and thrive in certain soils, and there is some reason to believe that it is indigenous to parts of South Africa. Few people will deny that it is to a large extent conveyed through the agency of contaminated water, but our home experience seems to show that it is communicable through other channels also, and that the protection of water, although of the highest importance to the public health, does not alone suffice to afford complete security. The protection of water in England has effected a notable reduction in the prevalence of enteric; has, in fact, brought it to a comparatively low level. But below that level it refuses to go, notwithstanding the protection which seems to indicate the potency of some other channel for the bacillus. Such considerations as these may well give pause to the more blatant critics of our military doctors, and might even be expected to teach them, if they were capable of learning, the existence of more things in heaven and earth than are dreamt of in their very limited philosophy.

It is certain that dust has been amongst the greatest of the nuisances with which our troops in Africa have had to contend, and there can be no doubt that dust would be extremely likely to become a vehicle of enteric contagion. Only those men who have learned the actual conditions under which the campaign must be carried on will be in a position to advise as to the means by which the health of the men can be preserved, and as to the extent to which these means are practicable. It is possible that the systematic destruction of infected materials by fire would be a useful measure of precaution. Whatever may happen it is useless, in the present state of knowledge, to expect immunity from fever or from dysentery; and we are afraid that the public must rest satisfied with knowing that those who occupy responsible positions are doing whatever is possible to minimise evils and dangers which cannot be altogether either suppressed or avoided—if only that knowledge can be obtained.

